# Relationships Between Work-to-Family Conflict and the Food Domain for Dual-Earner Parents With Adolescent Children

**DOI:** 10.3389/fpsyg.2021.752209

**Published:** 2021-12-16

**Authors:** Berta Schnettler, Edgardo Miranda-Zapata, Ligia Orellana, Héctor Poblete, Germán Lobos, Cristian Adasme-Berríos, María Lapo, Katherine Beroiza

**Affiliations:** ^1^Facultad de Ciencias Agropecuarias y Forestales, Universidad de La Frontera, Temuco, Chile; ^2^Núcleco Científico y Tecnológico de Biorecursos (BIOREN), Universidad de La Frontera, Temuco, Chile; ^3^Centro de Excelencia en Psicología Económica y del Consumo, Núcleo de Ciencias Sociales, Universidad de La Frontera, Temuco, Chile; ^4^Facultad de Especialidades Empresariales, Universidad Católica de Santiago de Guayaquil, Guayaquil, Ecuador; ^5^Facultad de Economía y Negocios, Universidad de Talca, Talca, Chile; ^6^Departamento de Economía y Administración, Universidad Católica del Maule, Talca, Chile

**Keywords:** work-family conflict, family meals, satisfaction with food-related life, dual-earner couples, adolescents

## Abstract

The impact of work-to-family conflict (WtoFC) can extend beyond family and work, and to other domains that contribute to well-being, such as the food domain. This study examined associations between WtoFC, perception of atmosphere of family meals (AFM), and satisfaction with food-related life (SWFoL) in dual-earner parents with adolescent children, and tested the mediating role of AFM between WtoFC and SWFoL. Questionnaires were administered to 473 different-sex dual-earner parents and one of their adolescent children (mean age 12.5 years, 51.4% male) in Temuco, Chile. Parents responded to a measure of work-to-family conflict; the three family members answered the Project-EAT Atmosphere of family meals scale, and the Satisfaction with Food-related Life Scale. Analyses were conducted using the Actor-Partner Interdependence Model and structural equation modeling. Results showed a negative association from WtoFC to SWFoL in both parents, while a more positive perception of atmosphere of family meals was linked to higher SWFoL in the three family members. Moreover, WtoFC was negatively linked to SWFoL in parents, while only mothers' WtoFC had a negative association with their adolescent children's SWFoL. Policymakers and organizations can contribute to workers' and their families' food-related well-being by fostering policies and measures to reduce WtoFC.

## Introduction

Work-to-family conflict (WtoFC) has a negative influence on other life domains besides work and family, such as health, and overall life satisfaction (Vieira et al., 2018; Yucel and Latshaw, 2020a,b; Matias and Recharte, 2021). One life domain that has been scarcely studied in this regard is food. Food acquisition, preparation, and consumption occupy an important part of an average person's life in terms of time, energy, and financial resources (Grunert et al., 2007; Schnettler et al., 2020b). There is evidence that job demands and WtoFC are related to unhealthy food consumption in workers from different countries (Djupegot et al., 2017; Liu et al., 2017). These negative outcomes may encompass more than nutritional issues, extending to the social dimension of food consumption (e.g., the perception of the atmosphere of meals, AFM) and the overall level of well-being in the food domain. Moreover, food consumption occurs in a social context (Schnettler et al., 2020a), and thus the impact of WtoFC on the food domain might extend beyond the individual to those in their close social environment. Therefore, this study focused on different-sex dual-earner parents with adolescent children, testing the influence of both parents' WtoFC on their own and on their adolescent child's SWFoL.

The influence of WtoFC on the food domain can be analyzed using the work-home resources (W-HR) model (ten Brummelhuis and Bakker, 2012). According to this model, conflict is a process whereby demands in one domain deplete personal resources, resulting in diminished outcomes in the other domain (ten Brummelhuis and Bakker, 2012). Food-related life, encompassing all food-related tasks and experiences, is part of the home sphere. In this line, satisfaction with food-related life (SWFoL) is defined as the person's overall cognitive assessment of their food and eating habits (Grunert et al., 2007). SWFoL has been positively associated with healthier eating habits, including frequent family meals, and other positive social outcomes such as family support and well-being in both adults and adolescents (Utter et al., 2018; Schnettler et al., 2021a,b). On this basis, following the WH-R model, it can be hypothesized that the impact of WtoFC might extend to the individual's SWFoL.

The social aspects of SWFoL (Schnettler et al., 2020b) suggest that the experiences comprised in this construct can have an interindividual impact. In accordance with family systems theory (Kerr and Bowen, 1988), evidence shows that family members sharing the same environment can influence one another's eating behaviors and SWFoL (Schnettler et al., 2020b). Adolescents' diet and food preferences are usually influenced by food environments, including the eating behaviors of their parents, meaning that adolescent children tend to develop eating behaviors like those of their family members (Piccoli et al., 2017; Patel et al., 2018; Fleary and Ettienne, 2019). The depletion of one's time and energy can make it difficult to engage positively and frequently in family activities, such as family meals, affecting the people with whom the individual is intimately connected (Lu et al., 2016).

**Figure 1:**
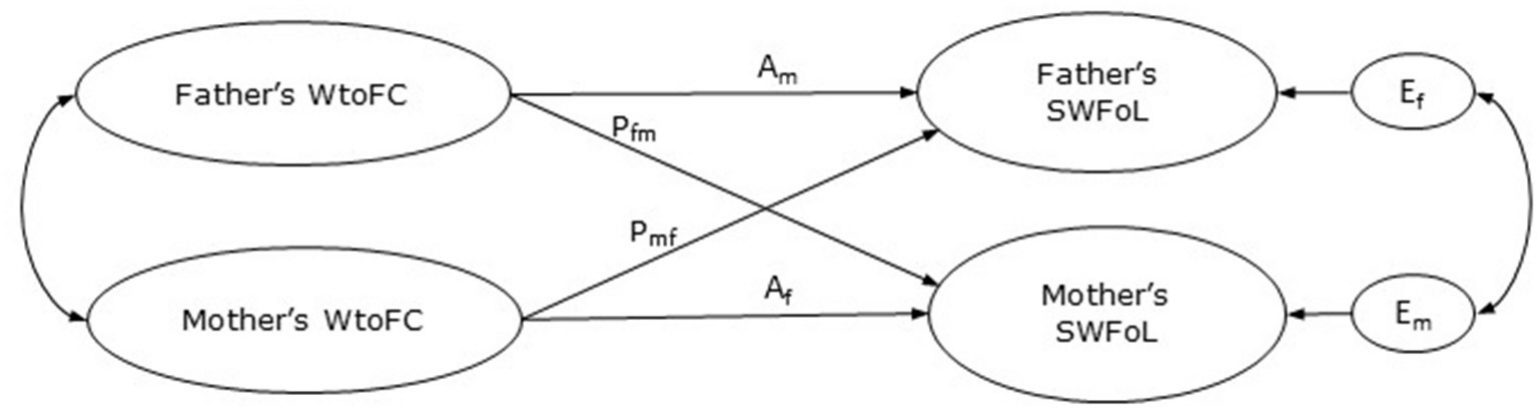
Basic actor-partner interdependence model of work-to-family conflict (WtoFC) and satisfaction with food-related life (SWFoL). A_m_, actor effect of father's WtoFC on his own SWFoL; A_f_, actor effect of mother's WtoFC on her own SWFoL; P_fm_, partner effect of father's WtoFC on mother's SWFoL; P_mf_, partner effect of mothers's WtoFC on fathers's SWFoL; E_f_ and E_m_, residual errors on SWFoL for the father and mother, respectively.

Negative crossover effects between members of a dyad (e.g., a couple, parent-child dyad) refers to the process by which a stressor or strain (e.g., job demands) experienced by one member of the dyad affect the other member's level of stress or strain (Westman, 2002). The Actor-Partner Interdependence Model (APIM, Kenny et al., 2006) allows to explore crossover effects between workers and their family members. In the APIM, actor effects are the outcomes predicted by the individuals' own characteristics, while partner effects are outcomes of one member of the dyad which are predicted by the characteristics of the other member (i.e., crossover, Kenny et al., 2006, see Figure 1 for a basic APIM design). However, as family meals not only involve both parents but also their children, and thus family meals are a family event (Cho and Allen, 2013; Utter et al., 2018; Schnettler et al., 2021a,b), this study used a triadic APIM approach to identify the effects among family variables, i.e., dyadic associations within a triadic design that include fathers, mothers and one adolescent children. Actor effects are observed when characteristics of one person are significant predictors of their own outcomes (i.e., parents' WtoFC on their own AFM and SWFoL, adolescents' AFM on their own SWFoL). Partner effects are observed when characteristics of one person in the family influence an outcome reported by another family member (i.e., mothers' WtoFC on fathers' AFM or SWFoL and vice versa, mothers' WtoFC on their adolescent children's AFM or SWFoL, fathers' WtoFC on their adolescent children's AFM or SWFoL, and adolescents' AFM on their parents' SWFoL).

Lastly, most research available on WtoFC, dual-earner couples and parent-child dyads have centered on families with young children and in developed countries (Le et al., 2020). Adolescence is a challenging period for parents' well-being, as it is a developmental period with distinctive needs and desires for both autonomy and support (Matias and Recharte, 2021), which extend to the food domain (Schnettler et al., 2018). Empirical evidence in this regard is still limited in Latin American countries, where also a traditional family structure with marked gender roles prevails for conducting food-related tasks (Orellana et al., 2021a; Schnettler et al., 2021b). The associations between parental WtoFC and children's well-being outcomes have yet to be explored in developing countries (Yucel and Latshaw, 2020b).

On this basis, this study focused on different-sex dual-earner parents with adolescent children in a developing country in Latin America, testing the interrelations between parents' WtoFC on their own and on their adolescent child's SWFoL. Following the W-HR model (ten Brummelhuis and Bakker, 2012), family systems theory (Kerr and Bowen, 1988), and using APIM framework (Kenny et al., 2006), the aims of this study were (1) to explore actor and partner effects between work-to-family conflict, perception of the atmosphere of family meals, and SWFoL in different-sex dual-earner parents with adolescent children, and (2) to explore whether the perception of the atmosphere of family meals has a mediating role between work-to-family conflict and SWFoL. Therefore, the perception of the atmosphere of family meals for the three family members was added to the APIM to test if their perception of the atmosphere of family meals has a mediating role between both parents' WtoFC and the three family members' SWFoL.

From the standpoint of the W-HR model, an individual dealing with work-home conflict faces excessive demands from the work domain, which drains their limited personal resources that otherwise would be directed toward fulfilling roles and demands in the home (ten Brummelhuis and Bakker, 2012). Diverse studies in Asian and European countries have shown that competing demands for workers' personal resources, such as time and energy, can result in negative outcomes in the food domain, mostly in terms of a lower-quality diet (Djupegot et al., 2017; Liu et al., 2017). These studies show that, for an individual, excessive demands from the work domain will prevent the investment of personal resources on healthier eating habits.

An individuals' WtoFC may also alter the person's relationship quality with their partner and children (ten Brummelhuis and Bakker, 2012). Studies on WtoFC in different-sex dual-earner couples have reported crossover effects from one partner's WtoFC to the other's well-being or domain or life satisfaction (Lu et al., 2016; Yucel and Latshaw, 2020a). Other studies have examined crossover effects in parent-child dyads. Higher WtoFC in mothers and fathers is linked to negative mental health and behavior outcomes in their children (Vieira et al., 2016; Dinh et al., 2017; Yucel and Latshaw, 2020b; Matias and Recharte, 2021), although mothers and fathers can have distinct effects in these outcomes. Fewer studies have focused on the effect on adolescent's domain satisfaction, but it has been reported (Orellana et al., 2021b), that both parents' WtoFC is indirectly associated with their adolescent children's family life satisfaction.

Eating habits associated with lower levels of SWFoL in adult and adolescent samples (Schnettler et al., 2018, 2020b, 2021b), such as having family meals less frequently, are more likely in parents with low balance between their work and home-related tasks (Djupegot et al., 2017). Given these findings, we argue that WtoFC may deplete personal resources that otherwise may be invested in the food domain, resulting in lower SWFoL in workers, their partner and adolescent children *via* crossover. Therefore, we posit the following hypotheses:

H1. Work-to-family conflict is negatively associated with satisfaction with food-related life for each parent (actor effects).
H2. Work-to-family conflict of one parent is negatively associated with (a) the other parent's, and (b) with the adolescent's satisfaction with food-related life (partner effects).

The daily conflicting work and school schedules of parents and their children (Cho and Allen, 2013; Sharif et al., 2017; Jones, 2018) allow few opportunities for all family members to spend time together throughout the day. Family meals thus become a convenient moment for the family to come together and interact, particularly during the evening meal. Beyond the act of feeding, family meals offer a space for parents and children to discuss conflicts, express affection, exchange information, values, support (Utter et al., 2018), and buffers against psychological stressors (Giray and Ferguson, 2018). For these positive exchanges to occur, however, a pleasant mealtime atmosphere is needed. Studies show that a positive mealtime atmosphere fosters higher well-being in family members (White et al., 2015; Giray and Ferguson, 2018; Jones, 2018; Utter et al., 2018). Family meals also have been found to increase parents' and their children's SWFoL (Schnettler et al., 2018, 2020a, 2021b). Thus, we posed the following hypotheses:

H3. The perception of the atmosphere of family meals is positively associated with satisfaction with food-related life for fathers, mothers, and adolescents (actor effects).
H4. The perception of the atmosphere of family meals of one parent is positively associated with (a) the other parent's, and (b) the adolescent's satisfaction with food-related life (partner effects).
H5. The perception of the atmosphere of family meals of adolescents is positively associated with their parents' satisfaction with food-related (partner effects).

Family meals can also be a source of tension, and parents experiencing WtoFC may avoid or shorten their family meals (Cho and Allen, 2013; Schnettler et al., 2021a). The preparation of family meals can be demanding because they involve multiple tasks, from planning, shopping, and cooking, to serving and cleaning up after the meal (Cho and Allen, 2013). Studies show that working parents may consider family meals as stressful events of their day, as it is their responsibility to fix this meal after coming home from work, or because they struggle to coordinate their own and their children's schedules and food preferences (Martin-Biggers et al., 2014). Moreover, the relational difficulties associated with WtoFC have been found mainly for mothers (Vieira et al., 2016; Matias et al., 2017; Matias and Recharte, 2021). Therefore, we argue that depletion of resources associated with one parent's WtoFC may deteriorate their own perception of the atmosphere of family meals, as well as that of their partner and adolescent children. Thus, we posed the following hypotheses:

H6. Work-to-family conflict is negatively associated with perception of the atmosphere of family meals for each parent (actor effects).
H7. Work-to-family conflict of one parent is negatively associated with (a) the other parent's, and (b) the adolescent's perception of the atmosphere of family meals (partner effects).

The negative effects of WtoFC may not always be direct. Research provides evidence of variables mediating between individuals' WtoFC and their family members' outcomes (e.g., Wang and Peng, 2017; Matias and Recharte, 2021; Orellana et al., 2021b). Mediators from the food domain have been scarcely considered, although family meals have shown a mediating role between long work hours and positive family outcomes (Jones, 2018). Therefore, we posed the last hypotheses as follow:

H8. The perception of the atmosphere of family meals has a mediating role between both parents' work-to-family conflict and satisfaction with food-related life for the three family members (actor and partner effects).

Lastly, differences in WtoFC effects according to the parent's gender are expected (Yucel and Latshaw, 2020b). Caro et al. (2017) reported that children perceive their mother's work, more than their father's work, as a source of tension that affects the family dynamic. This perception may derive from upholding traditional family gender roles, in which mothers spend more time on childcare and housework than fathers, whereas fathers complete more hours on paid work than mothers (Yucel and Latshaw, 2020b). Food is a relevant domain in which to explore the effects of WtoFC as all food-related tasks in the household are considered the mother's responsibility (Persson Osowski and Mattsson Sydner, 2019; Schnettler et al., 2021b), with stronger demands in lower gender-equity contexts, such as Latin American regions (Schnettler et al., 2021b). Furthermore, daily food-related chores and parenting practices can be sources of disagreement and arguments between parents and adolescents (Martin-Biggers et al., 2014; Matias and Recharte, 2021). This parent-child bickering may become more pronounced when the mother's personal resources are directed to respond to work demands (Matias and Recharte, 2021). As the main responsibility for family meals is attributed to women, the associations between WtoFC, the atmosphere of family meals and SWFoL may be different according to the parent's gender.

## Methods

### Participants and Procedure

This study had a non-probabilistic sample of 473 different-sex dual-earner parents with at least one child aged between 10 and 15 years, living in Temuco, Chile. Mother, father, and adolescent responded to a questionnaire. Sample characteristics are displayed in Table 1. This study is part of a longitudinal research that examines the interdependence between the job, family, and food domains in Chilean families (Schnettler et al., 2018). Sample size was determined considering 10 participants for each item of each scale used in this project, accounting for 30% of sample mortality as the study progresses.

**Table 1:** Sample characteristics (*n* = 473).

**Characteristic**	**Total sample**	***P*-value^a^**
**Age [Mean (*SD*)]^a^**		
Mother	39.1 (7.2)	<0.001
Father	42.0 (8.9)	
Adolescents	12.5 (1.7)	
**Adolescents' gender (%)**		
Male	51.4	
Female	48.6	
Number of family members [Mean (*SD*)]	4.4 (1.0)	
Number of children [Mean (*SD*)]	2.2 (0.8)	
**Socioeconomic status (%)**		
High	22.2	
Middle	61.5	
Low	16.3	
**Gender of the main breadwinner (%)**		
Female	23.3	
Male	76.7	
**Number of days/week couples ate together [Mean (** ***SD*** **)]**		
Breakfast	2.8 (2.3)	
Lunch	3.3 (2.2)	
Supper	5.1 (2.5)	
Dinner	2.5 (3.1)	
**Number of days families eat different types of foods [Mean (SD)]**		
Homemade foods	6.1 (1.5)	
Buy ready-to eat food	0.6 (1.1)	
Order food at home	0.6 (0.7)	
Eat at restaurants	0.3 (0.7)	
Eat at fast-food outlets	0.4 (0.7)	
**Number of hours per day spent cooking during the week [Mean (SD)]^b^**		
Mother	2.2 (1.3)	<0.001
Father	1.0 (1.3)	
Other person	0.9 (1.6)	
**Number of hours per day spent cooking on the weekend [Mean (SD)]^b^**		
Mother	3.0 (1.7)	<0.001
Father	1.6 (1.5)	
Other person	0.6 (1.3)	
**Type of employment (%)** ^**c**^		
Woman employee	72.7	
Woman self-employed	27.3	0.460
Man employee	74.8	
Man self-employed	25.2	
Working hours (%)^c^		
Woman working 45 h per week	59.2	<0.001
Woman <45 h per week	40.8	
Man working 45 h per week	72.3	
Man working <45 h per week	27.7	

a *Independent sample t-test*.

b *Analysis of variance*.

c *P-value corresponds to the (bilateral) asymptotic significance obtained in Pearson's Chi-square Test*.

Families were recruited from schools of socioeconomically diverse backgrounds in the city. Interviewers contacted parents to introduce the study, its aims and confidential and anonymized treatment of information, and the structure of the questionnaire. Families were visited in their homes by the interviewers. Parents signed written informed consent and the adolescents signed assent forms, and then interviewers administered each questionnaire separately to the three family members. After the questionnaires were responded, each family received a gift card worth approximately 15 USD. Data was collected between August and December 2019 and registered in the online survey platform QuestionPro (QuestionPro Inc.).

The recruitment and data collection method above was first pilot tested with 20 families, and no changes were required in the procedure nor in the questionnaire. The Ethics Committee of the University of La Frontera approved this study.

### Measures

#### Instruments Answered by Mothers and Fathers

**Work-to-family conflict (WtoFC)**. Four items were used related to the negative influence of work on the family, adapted from the work of Frone et al. (1992), and Netemeyer et al. (1996, see Kinnunen et al., 2006). In these items, time or strain are the mechanisms by which spillover occurs from the work to the family domain (e.g., “Does your job or your career keep you from spending the time that you'd wish with your family?”). The Spanish version of this dimension was used (Orellana et al., 2021b). Each item was responded on a 5-point Likert scale (1: “never;” 5: “very often”). WtoFC scores were obtained by summing up the four items, with higher scores representing higher WtoFC. In this study, the standardized factor loadings of the work-to-family conflict dimension ranged from 0.707 to 0.919 for mothers and from 0.707 to 0.965 for fathers, all statistically significant (*p* < 0.01). The average extracted variance (AVE) values were higher than 0.50 (AVE mothers = 0.73, fathers = 0.75). The work-to-family conflict measure showed good internal reliability, as the Omega coefficient was 0.91 for mothers and 0.92 for fathers.

#### Instruments Answered by the Three Family Members

**Project-EAT atmosphere of family meals** (AFM, Neumark-Sztainer et al., 2004). This four-item scale assesses mealtime atmosphere, with two items related to enjoyment of mealtimes (e.g., “I enjoy eating meals with my family”) and two items about mealtime communication (e.g., “In my family, dinner/supper time is about more than just getting food, we all talk with each other”). The Spanish version of the AFM scale was used (Schnettler et al., 2018). In Chile, supper is traditionally chosen instead of dinner, and it is an evening meal to be shared with others, hence it was included in the last item alongside dinner. Respondents indicated their degree of agreement with each statement using a 6-point Likert scale (1: completely disagree; 6: completely agree). AFM scores were obtained by summing up the scores from the four items, with higher scores representing a more positive mealtime atmosphere. The AFM showed a good internal consistency in a study conducted in the UK (White et al., 2015). In the present study, the standardized factor loadings of the AFM scale ranged from 0.851 to 0.949 for mothers, from 0.802 to 0.918 for fathers and from 0.798 to 0.903 for adolescents, all statistically significant (*p* < 0.01). The average extracted variance (AVE) values were higher than 0.50 (AVE mothers = 0.78, fathers = 0.74, adolescents = 0.71). The AFM scale showed good internal reliability, as Omega coefficient was 0.93 for mothers, 0.92 for fathers and 0.91 for adolescents.

**Satisfaction with food-related Life** (SWFoL, Grunert et al., 2007). This is a five-item scale measuring a person's overall assessment of their food and eating habits (e.g. “Food and meals are positive elements”). The Spanish version of the SWFoL was used, which has shown good internal consistency in Chilean adult, adolescent and dual-earner parent samples (e.g., Schnettler et al., 2021a,b). Respondents indicated their degree of agreement with each statement using a 6-point Likert scale (1: completely disagree; 6: completely agree). SWFoL scores were obtained by summing up the five items, with higher scores representing higher SWFoL. In this study, the standardized factor loadings of the SWFoL scale ranged from 0.707 to 0.867 for mothers, from 0.752 to 0.868 for fathers and from 0.493 to 0.840 for adolescents, all statistically significant (*p* < 0.01). The AVE values were higher than 0.50 (AVE mothers = 0.62, fathers = 0.66, adolescents = 0.52). The scale showed good internal reliability, as Omega coefficient was 0.89 for mothers, 0.90 for fathers and 0.84 for adolescents.

#### Supplementary Questions

The three family members were asked about their age; adolescents were asked about their gender. Parents were asked about their type of employment and the number of working hours per week. Women were asked about the number of family members, the number of children, the gender of the person with the highest income in the household, the number of days that all family members eat together during the week (separately for breakfast, lunch, supper and dinner); the number of days per week that they eat homemade food, buy ready-to-eat food, order food at home, or eat at restaurants or fast-food outlets; and the number of hours per day that they, their male partner and other person spent cooking during the week and on weekend. The socioeconomic status (SES) was determined based on the total household income and its size [Asociación de Investigadores de Mercado (AIM), 2016].

### Statistical Analysis

For descriptive analysis, SPSS v. 23 was used. In order to test measurement invariance of instruments, we follow Claxton et al. (2015) statements. Thus, a confirmatory factor analysis (CFA) was used to test the invariance of the Work-to-family conflict measure among parents and the invariance of the Project-EAT Atmosphere of Family Meals Scale and the Satisfaction with Food-related life Scale among the three family members. Configural invariance, metric invariance, and scalar invariance were verified (Dimitrov, 2010).

To test the first seven hypotheses, we used an extended version of the actor-partner interdependence model (APIM) to examine intrapersonal (actor) effects within family members and interpersonal (partner) effects between family members. Our analytical strategy uses a triadic APIM approach using structural equation modeling (SEM) with distinguishable triads. This approach facilitates the assessment of the extent to which members of a family influence each other and testing specific hypotheses at different levels. This approach was used by Weidmann et al. (2018) with triads composed by both parents and one child. In the present study, the effects between both parents' work-to-family conflict (WtoFC), and the three family members' perception of the atmosphere of family meals (AFM) and satisfaction with food-related life (SWFoL) were tested. Actor effects are those associations between one parent's WtoFC, AFM, and SWFoL and the associations between AFM and SWFoL for mother, father, and adolescent. Although the present study has a triadic approach, partner effects are traditionally understood as dyadic associations within the dyads mother-father and parent-adolescent, i.e., the associations between one parent's WtoFC and the other parent's AFM and SWFoL, the associations between each parent's WtoFC and the adolescent's AFM and SWFoL and, the associations between adolescent's AFM and each parent's SWFoL.

The APIM controls for the extent to which each parent's WtoFC affects the other's by establishing a correlation between independent variables for each dyad member. The APIM also includes correlations between the residual errors of the dependent variables of each family member (i.e., father's, mother's, and adolescent's SWFoL), which controls for other sources of interdependence between actors and partners (Kenny et al., 2006).

Variables with direct effects on the dependent variables of three family members (i.e., AFM and SWFoL) were incorporated in the analysis to control for their effects when modeling the fit of the data. These variables were parent's age, type of employment and their number of working hours as well as the family SES, the number of children, and the number of days a week in which all family members ate supper together.

CFA and SEM were conducted using Mplus 7.11. CFA and Structural model parameters were estimated using the robust unweighted least squares (ULSMV). Considering the ordinal scale of the items, the SEM analysis was done *via* the polychoric correlation matrix. The model Chi-square (χ^2^), Tucker-Lewis index (TLI), the comparative fit index (CFI) and the root mean square error of approximation (RMSEA) determined the model fit of the data. A good model fit is found when the model χ^2^ is non-significant. The TLI and CFI showed a good fit with a value above 0.95. A good fit is defined as the value of the RMSEA being below 0.06 (Hu and Bentler, 1999).

To determine the achievement of configural invariance of the Work-to-family conflict measure, the Project-EAT Atmosphere of Family Meals Scale and the Satisfaction with Food-related life Scale, and, the same fit indices described for the SEM were used. To establish the achievement of metric and scalar invariance, CFI differences (D_CFI) were used. A D_CFI lower than −0.01 indicates that invariance was not meet (Cheung and Rensvold, 2002).

Lastly, the proposed mediating role of each family member's AFM between both parents' WtoFC and SWFoL for the three family members (hypothesis 8) was examined using the Actor-partner interdependence mediation model. For instance, an intrapersonal association comprised to test the mediating role of the mothers' AFM between their own WtoFC and SWFoL, the same for the fathers (i.e., mothers' WtoFC → mothers' AFM → mothers' SWFoL and fathers' WtoFC → fathers' AFM → fathers' SWFoL). Inter-individual associations comprised to test the mediating role of one parent's AFM between the other parent's WtoFC and SWFoL (i.e., mothers' WtoFC → fathers' AFM → mothers' SWFoL and fathers' WtoFC → mothers' AFM → fathers' SWFoL), and the mediating role of the adolescents' AFM between each one of their parents WtoFC and SWFoL (i.e., mothers' WtoFC → adolescents' AFM → mothers' SWFoL and fathers' WtoFC → adolescents' AFM → fathers' SWFoL). The mediating role of each parent' AFM also was tested between each parent's WtoFC and the adolescents' SWFoL (i.e., mothers' WtoFC → mothers' AFM → adolescents' SWFoL and fathers' WtoFC → fathers' AFM → adolescents' SWFoL). Lastly, the mediating role of the adolescents' AFM was tested between each parent's WtoFC and the adolescents' SWFoL (i.e., mothers' WtoFC → adolescents' AFM → adolescents' SWFoL and fathers' WtoFC → adolescents' AFM → adolescents' SWFoL). SEM was also used to test the mediating role of AFM through a bias-corrected (BC) bootstrap confidence interval using 1,000 samples (Lau and Cheung, 2012). When the resulting BC confidence interval did not include zero, the mediating role was supported.

## Results

### Sample Description

Sociodemographic characteristics of the sample encompassing 473 families of mothers, fathers and adolescents are shown on Table 1. On average, mothers were 39.1 years old, fathers 42.0 years, and adolescents 12.5 years. In the latter group, 48.6% were female. The difference between mothers' and fathers' age was significant (*p* < 0.001). Most families had a middle SES and the person with a higher income in the household was a man. Families were composed of an average of four family members and two children.

Families ate together breakfast, lunch, and dinner few days per week, but consumed homemade food frequently. Mothers spent a significantly higher number of hours per day cooking during the week and on weekends, compared to their male partners and other persons (*p* < 0.001). During the week, the average number of hours per day that fathers and other persons spent cooking did not differ from each other, while on weekends fathers had a significantly higher average number of hours per day that they spent cooking than other persons. “Other persons” mainly include grandmothers and adolescent children. Most of the parents in this sample—women and men—had a full-time job (45 hours per week in Chile). Compared to mothers, there was a greater proportion of fathers who worked full time (*p* < 0.001). The proportions of employed vs. independent workers did not differ between mothers and fathers (*p* > 0.1).

Table 2 shows the average scores and correlations for work-to-family conflict (WtoFC), perception of the atmosphere of family meals (AFM) and satisfaction with food-related life (SWFoL). Most of the correlations were significant and in the expected directions, except for those between mother's WtoFC and both their own and the father's AFM; and between the father's WtoFC and their own, the mother's and the adolescent's AFM, as well as with the adolescent's SWFoL. Mothers and fathers did not differ in the average scores for WtoFC (*t* = 1.067, *p* = 0.286) nor in the average score of each item of the WtoFC measure used in this study. Adolescents scored significantly lower than their mothers and fathers in AFM (F = 6.299, *p* = 0.002), whereas both parents did not differ from one another. Adolescents scored significantly higher than their mothers and fathers in the SWFoL (*F* = 26.463, *p* < 0.001), and fathers scored significantly higher than mothers.

**Table 2:** Descriptive statistics and correlations for parent's Work-to-Family Conflict (WtoFC) and the three family members' Atmosphere of Family Meals (AFM), and Satisfaction with Food-related Life (SWFoL) in dual-earner parents with adolescent children (*n* = 473).

	**M (SD)**	**Correlations**							
		**1**	**2**	**3**	**4**	**5**	**6**	**7**	**8**
1. Mother's WtoFC	10.63 (4.49)	–	0.146^**^	−0.040	−0.036	−0.096^*^	−0.140^**^	−0.101^*^	−0.103^**^
2. Father's WtoFC	10.32 (2.46)		1	−0.047	−0.049	−0.044	−0.094^*^	−0.129^**^	−0.039
3. Mother's AFM	21.67 (3.24)			1	0.437^**^	0.258^**^	0.294^**^	0.274^**^	0.241^**^
4. Father's AFM	21.26 (3.49)				1	0.245^**^	0.201^**^	0.422^**^	0.236^**^
5. Adolescent's AFM	20.88 (3.54)					1	0.112^*^	0.161^**^	0.328^**^
6. Mother's SWFoL	21.32 (4.83)						1	0.380^**^	0.308^**^
7. Father's SWFoL	22.41 (5.01)							1	0.243^**^
8. Adolescent's SWFoL	23.58 (4.50)								1

* *Correlation is significant at the 0.05 level (2-tailed)*.

** *Correlation is significant at the 0.01 level (2-tailed)*.

### Measurement Invariance

Supplementary Tables 1–7 show the results of the CFA models in the Work-to-Family Conflict (WtoFC) measure among both parents, and in the Project-EAT Atmosphere of family meals (AFM) and the Satisfaction with Food-related life (SWFoL) scale among both parents, among mothers and their adolescent children as well as among fathers and their adolescent children. All models achieved configural and metric invariance. Scalar invariance was achieved only for WtoFC and AFM among mothers and fathers.

### APIM Results: Testing Actor-Partner Hypotheses

**Figure 2:**
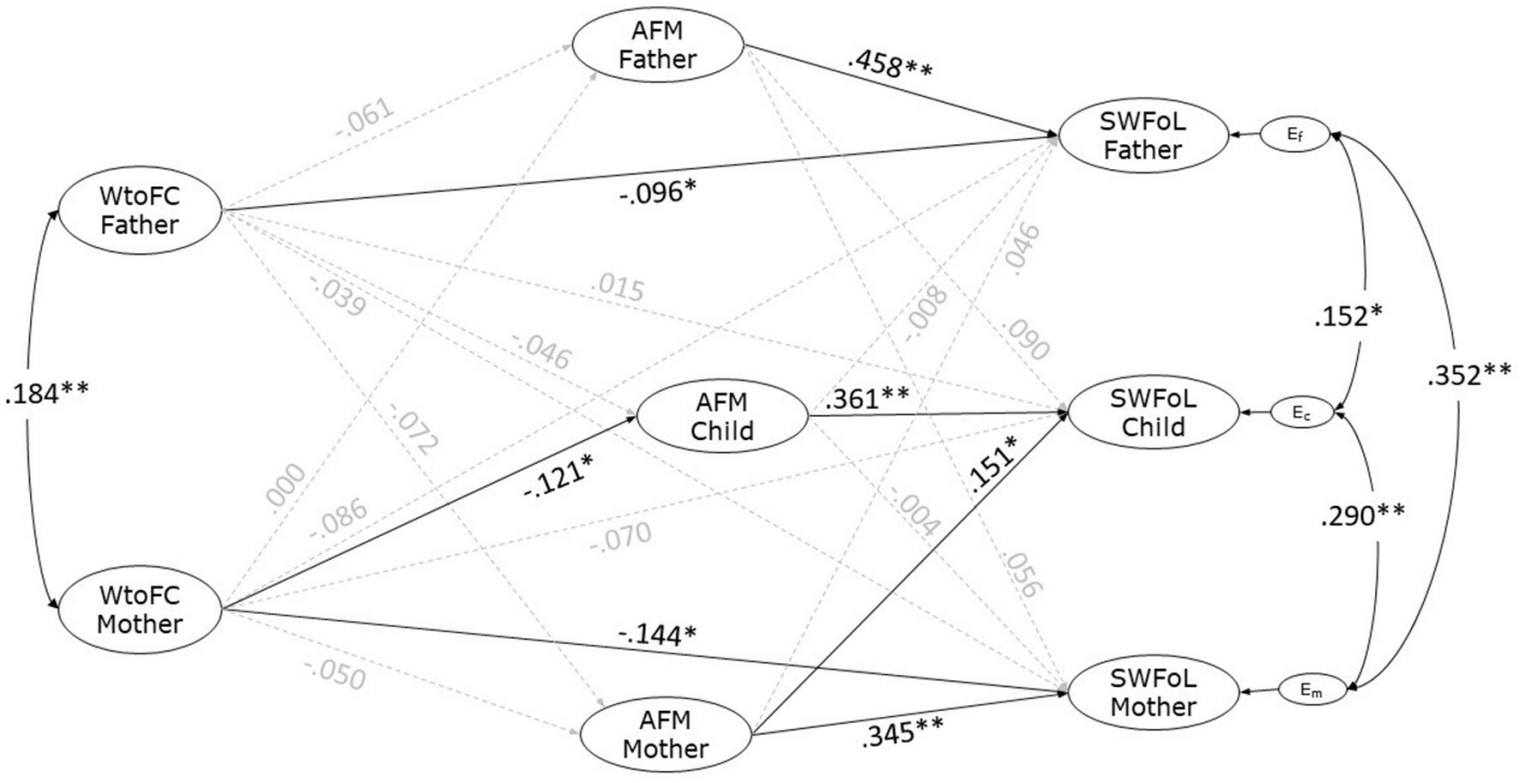
Actor-partner interdependence model of the effect of parent's Work-to-Family Conflict (WtoFC) on the three family members' Perception of the Atmosphere of Family Meals (AFM) and Satisfaction with Food-related Life (SWFoL) in dual-earner parents with adolescent children. E_f_, E_c_ and E_m_, residual errors on SWFoL for the fathers, mothers, and their adolescent children, respectively. *p < 0.05; **p < 0.01. The control for the effects both members of the couple's age, type of employment and their number of working hours as well as the family SES, the number of children and the number of supper meals in which all the family members ate together during a week on the dependent variables of the three family members (AFM and SWFoL) were not shown in the path diagram.

The results from the estimation of the structural model are shown in Figure 2. The model had an acceptable fit with the data [$\chi_{\left(793,\;\text{n}\;=\;473\right)}^{2}$ = 1037.489, *p* < 0.001; CFI = 0.942; TLI = 0.931; RMSEA = 0.026; 90% CI = 0.021, 0.030]. Although the χ^2^-test was significant, it is well-established that the χ^2^-test is sensitive to sample size (Cheung and Rensvold, 2002), as is the case in this study. A significant correlation (covariance) was found between both parents' WtoFC (*r* = 0.184, *p* < 0.001). Significant correlations were also found between the residual errors of mother's and father's SWFoL (*r* = 0.352, *p* < 0.001), between mother's and adolescent's SWFoL (*r* = 0.290, *p* < 0.001) as well as between father's and adolescent's SWFoL (*r* = 0.152, *p* = 0.015).

H1 stated that the work-to-family conflict is negatively associated with SWFoL for each parent. As shown in Figure 2, the path coefficients (standardized) indicate that the father's WtoFC was negatively associated with his own SWFoL (γ = −0.096, *p* = 0.018). Similarly, the mother's WtoFC was negatively associated with her own SWFoL (γ = −0.144, *p* = 0.002). These findings supported H1.

H2 stated that work-to-family conflict of one parent is negatively associated with (a) the other parent's and (b) with the adolescent's SWFoL (partner effects). Results showed that the father's WtoFC was not statistically associated with the mother's SWFoL (γ = −0.039, *p* = 0.373), nor the mother's WtoFC was statistically associated with the father's SWFoL (γ = −0.086, *p* = 0.056). The father's (γ = 0.015, *p* = 0.728) and mother's (γ = −0.070, *p* = 0.160) WtoFC were not significantly associated with the adolescent's SWFoL. Therefore, H2 was not supported.

Following H3, it was expected that perception of the atmosphere of family meals is positively associated with SWFoL for fathers, mothers and adolescents. The path coefficients indicate that perception of the atmosphere of family meals was positively associated with SWFoL for the father (γ = 0.458, *p* < 0.001), the mother (γ = 0.345, *p* < 0.001), and their adolescent child (γ = 0.361, *p* < 0.001). These findings supported H3.

H4 stated that the perception of the atmosphere of family meals of one parent is positively associated with (a) the other parent's, and (b) the adolescent's SWFoL. For H4a, it was found that the father's perception of the atmosphere of family meals was not significantly associated with the mother's SWFoL (γ = 0.056, *p* = 0.380). Likewise, the mother's perception of the atmosphere of family meals was not significantly associated with the father's SWFoL (γ = 0.046, *p* = 0.399). For H4b, results showed that the father's perception of the atmosphere of family meals was not significantly associated with the adolescent's SWFoL (γ = 0.090, *p* = 0.137), whereas the mother's perception of the atmosphere of family meals was positively associated with the adolescent's SWFoL (γ = 0.151, *p* = 0.022). These findings thus H4 partially, H4a for parents, was not supported, while H4b was partially supported, for mothers and adolescents.

Also related to family meals, H5 stated that the perception of this atmosphere in adolescents is positively associated with their parents' SWFoL. These associations were not significant for neither dyad father-adolescent (γ = −0.008, *p* = 0.896) not mother-adolescent (γ = −0.004, *p* = 0.949). Hence, H5 was not supported.

H6 stated that WtoFC is negatively associated with the perception of the atmosphere of family meals for each parent. The path coefficients indicate that WtoFC was not significantly associated with the perception of the atmosphere of family meals, neither for fathers (γ = −0.061, *p* = 0.230) nor for mothers (γ = −0.050, *p* = 0.372). These findings did not support H6.

H7 stated that WtoFC of one parent is negatively associated with (a) the other parent's and (b) the adolescent's perception of the atmosphere of family meals. Results for H7a indicate that the father's WtoFC was not significantly associated with the mother's perception of the atmosphere of family meals (γ = −0.072, *p* = 0.065), nor was the mother's WtoFC significantly associated with the father's perception of the atmosphere of family meals (γ = 0.000, *p* = 1.000). For H7b, the father's WtoFC was not significantly associated with the adolescent's perception of the atmosphere of family meals (γ = −0.046, *p* = 0.338). By contrast, the mother's WtoFC was negatively associated with the adolescent's perception of the atmosphere of family meals (γ = 0.121, *p* = 0.017). These findings did not support H7a, while H7b was partially supported for mothers and adolescents.

Most of the control variables did not affect the model significantly (Table 3). The father's type of employment (employee vs. independent) positively affected their own SWFoL (γ = 0.138, *p* < 0.05). The mother's working hours negatively affected the adolescents SWFoL (γ = −0.116, *p* < 0.05). The father's type of employment (employee vs. independent) negatively affected the mother's perception of the atmosphere of family meals (γ = −0.137, *p* < 0.05). The mother's age negatively affected the adolescent's perception of the atmosphere of family meals (γ = −0.149, *p* < 0.05), and a similar result was found for family SES (γ = −0.195, *p* < 0.01).

**Table 3:** Standardized effects estimate of control variables on Satisfaction with Food-related life (SWFoL) and atmosphere of family meals (AFM) in dual-earner parents with adolescent children.

	**Estimate**	***p*-value**
Mother's age → Mother's SWFoL.	0.060	0.433
Mother's type of employment → Mother's SWFoL	0.049	0.378
Mother's working hours → Mother's SWFoL	−0.040	0.469
Father's age → Mother's SWFoL	0.006	0.941
Father's type of employment → Mother's SWFoL	0.047	0.373
Father's working hours → Mother's SWFoL	−0.030	0.553
Family socioeconomic status → Mother's SWFoL	−0.030	0.580
Number of children → Mother's SWFoL	−0.037	0.463
Number of supper times per week ate together → Mother's SWFoL	0.012	0.804
Mother's age → Father's SWFoL	0.040	0.422
Mother's type of employment → Father's SWFoL	0.013	0.841
Mother's working hours → Father's SWFoL	−0.068	0.212
Father's age → Father's SWFoL	−0.006	0.909
Father's type of employment → Father's SWFoL	0.138	0.030
Father's working hours → Father's SWFoL	0.013	0.779
Family socioeconomic status → Father's SWFoL	−0.091	0.051
Number of children → Father's SWFoL	−0.035	0.490
Number of supper times per week ate together → Father's SWFoL	0.082	0.102
Mother's age → Adolescent's SWFoL	0.012	0.803
Mother's type of employment → Adolescent's SWFoL	0.029	0.702
Mother's working hours → Adolescent's SWFoL	−0.116	0.034
Adolescent's age → Adolescent's SWFoL	−0.001	0.984
Adolescent's type of employment → Adolescent's SWFoL	−0.025	0.733
Adolescent's working hours → Adolescent's SWFoL	−0.043	0.417
Family socioeconomic status → Adolescent's SWFoL	−0.046	0.347
Number of children → Adolescent's SWFoL	0.009	0.872
Number of supper times per week ate together → Adolescent's SWFoL	0.020	0.677
Mother's age → Mother's AFM.	−0.013	0.875
Mother's type of employment → Mother's AFM	−0.062	0.273
Mother's working hours → Mother's AFM	0.011	0.847
Father's age → Mother's AFM	0.020	0.809
Father's type of employment → Mother's AFM	−0.137	0.027
Father's working hours → Mother's AFM	0.021	0.723
Family socioeconomic status → Mother's AFM	−0.012	0.823
Number of children → Mother's AFM	0.005	0.926
Number of supper times per week ate together → Mother's AFM	0.075	0.171
Mother's age → Father's AFM	−0.009	0.906
Mother's type of employment → Father's AFM	−0.006	0.921
Mother's working hours → Father's AFM	0.022	0.707
Father's age → Father's AFM	0.014	0.860
Father's type of employment → Father's AFM	−0.093	0.092
Father's working hours → Father's AFM	−0.004	0.947
Family socioeconomic status → Father's AFM	0.040	0.492
Number of children → Father's AFM	−0.023	0.680
Number of supper times per week ate together → Father's AFM	0.082	0.149
Mother's age → Adolescent's AFM	−0.149	0.045
Mother's type of employment → Adolescent's AFM	−0.081	0.135
Mother's working hours → Adolescent's AFM	−0.034	0.544
Adolescent's age → Adolescent's AFM	−0.032	0.674
Adolescent's type of employment → Adolescent's AFM	−0.035	0.514
Adolescent's working hours → Adolescent's AFM	−0.037	0.481
Family socioeconomic status → Adolescent's AFM	−0.195	0.001
Number of children → Adolescent's AFM	0.073	0.155
Number of supper times per week ate together → Adolescent's AFM	−0.049	0.347

### Testing Mediating Roles of the Perception of the Atmosphere of Family Meals

The last hypothesis tested the mediating role of the perception of the atmosphere of family meals between both parents' WtoFC and SWFoL for the three family members (H8). The role of the adolescent's perception of the atmosphere of family meals as a mediator in the relationship between the mother's WtoFC and the adolescent's SWFoL was supported by a significant indirect effect obtained with the bootstrapping confidence interval procedure (standardized indirect effect = −0.046, 95% CI = −0.008, −0.005), as the confidence intervals did not include zero. No other indirect effect of the perception of the atmosphere of family meals was found because these confidence intervals did include zero (Table 4). These findings partially supported the mediating role of PAFM between both parents' work-to-family enrichment and the three family members' SWFoL.

**Table 4:** Bias-corrected confidence intervals of specific mediation effects of the three family members' perception of the atmosphere of family meals (AFM).

**Effects**	**Lower 2.5%**	**Estimate**	**Upper 2.5%**
From mothers' WtoFC to mothers' SWFoL Specific Indirect			
Mothers' SWFoL			
Mothers' AFM			
Mothers' WtoFC	−0.061	−0.019	0.023
Mothers' SWFoL			
Fathers' AFM			
Mothers' WtoFC	−0.006	−0.000	0.006
Mothers' SWFoL			
Adolescents' AFM			
Mothers' WtoFC	−0.016	0.001	0.017
From fathers' WtoFC to fathers' SWFoL Specific Indirect			
Fathers' SWFoL			
Mothers' AFM			
Fathers' WtoFC	−0.014	−0.004	0.006
Fathers' SWFoL			
Fathers' AFM			
Fathers' WtoFC	−0.083	−0.032	0.020
Fathers' SWFoL			
Adolescents' AFM			
Fathers' WtoFC	−0.006	0.000	0.007
From mothers' WtoFC to adolescents' SWFoL Specific Indirect			
Adolescents' SWFoL			
Mothers' AFM			
Mothers' WtoFC	−0.026	−0.008	0.010
Adolescents' SWFoL			
Adolescents' AFM			
Mothers' WtoFC	−0.008	−0.046	−0.005
From fathers' WtoFC to adolescents' SWFoL Specific Indirect			
Adolescents' SWFoL			
Fathers' AFM			
Fathers' WtoFC	−0.018	−0.006	0.006
Adolescents' SWFoL			
Adolescents' AFM			
Fathers' WtoFC	−0.054	−0.018	0.019

*WtoFC, Work-to-family conflict; SWFoL, Satisfaction with food-related life*.

## Discussion

This study shows that workers experiencing depletion of resources derived from job demands can also experience associated outcomes, for themselves and their families, in the food domain. The findings showed negative low strength relationships between work-to-family conflict and satisfaction with food-related life regardless of the parent's gender. Parents' WtoFC was not associated with their perception of the atmosphere of family meals, but a more positive perception of the atmosphere of family meals was linked to higher SWFoL showing medium strength relationships in both parents and their adolescent children. Furthermore, only the mothers' perception of the atmosphere of family meals was positively associated with their adolescent children's SWFoL by a low strength relationship; and only the mothers' WtoFC was indirectly associated with their adolescent children's SWFoL through the adolescent's perception of the family meal atmosphere, again, by a low strength indirect effect. Nevertheless, these results underscore the major role that mothers have on family dynamics, including family meals, reinforcing the relevance that mothers' management of their work and family roles have on mother-child interaction and on their children well-being in specific life domains.

### Actor Effects

Intra-individual associations were examined between the variables under study, that is, actor effects. It was expected that a higher WtoFC would be linked to lower satisfaction in the food domain, for both mothers and fathers. Our results suggest that depletion of personal resources in the work domain may compromise a parent's capacity for performing household chores, including food-related tasks (ten Brummelhuis and Bakker, 2012); the latter, in turn, decreases the parent's SWFoL. Specifically, workers who face competing demands for their time and energy may have a lower diet quality (Djupegot et al., 2017; Liu et al., 2017), use emotion-focused food choices (e.g., quick and easy food) as a cooping strategy when experience WtoFC (Devine et al., 2006; Cho and Allen, 2013), or may serve less healthful meals to their families, in the case of parents (Djupegot et al., 2017). This lower engagement with the food domain can result in the decrease of SWFoL in adults, regardless of gender (Schnettler et al., 2020a, 2021b). Regardless of the above, it should be noted that both actor effects were of low strength.

Furthermore, the more pleasant the atmosphere of family meals was perceived by parents and adolescents, the higher the levels of SWFoL they would experience. This is in line with other studies linking pleasant family meals to parental and child well-being (White et al., 2015; Giray and Ferguson, 2018; Jones, 2018; Utter et al., 2018), and to higher SWFoL (Schnettler et al., 2020a, 2021a). Moreover, the three partner effects found here were of medium strength, suggesting that both parents and adolescents have a similar AFM → SWFoL relationship. This finding supports previous qualitative evidence showing that mothers, fathers, and adolescents coincided in including family meals as part of their SWFoL (Schnettler et al., 2020a).

Other actor effects referred to negative associations between each parent's WtoFC and their own perception of the atmosphere of family meals. Contrary to this expectation (Vieira et al., 2016), this relationship was non-significant for both mothers and fathers. One possible explanation may be associated with the findings reported by Cho and Allen (2013), who did not find support for a buffer effect of family meals atmosphere against WtoFC in working parents, i.e., those workers who have a pleasant perception of family meal time did not differ from those workers who reported a negative perception, in terms of family meals frequency. In contrast with the W-HR model, our results suggest instead that job demands and strains do not take away the personal resources that a parent may invest in this social aspect of food consumption (i.e., their capacity to enjoy and communicate during family mealtimes).

Gender dynamics were also observed in actor effects. First, fathers and mothers did not differ in their WtoFC scores. This result was unexpected, as studies from European countries show that women in dual-earner couples are more likely to be affected by work-to-family conflict than their male partners (Vieira et al., 2018). A second gender-specific result was that, while the WtoFC → SWFoL association was of similar strength (following Cohen, 1988) in both parents, mothers showed significantly lower SWFoL than fathers. It can be hypothesized that, for women, WtoFC undermines their fulfillment of gender expectations, as food-related tasks and family meals are predominantly attributed to women (Persson Osowski and Mattsson Sydner, 2019; Schnettler et al., 2021b). This trend is also reflected in the result showing that both mothers and fathers in this sample cooked during the week, but mothers spent significantly more hours on this activity than fathers.

### Partner Effects

Another set of effects tested whether an individual's WtoFC can have consequences on outcomes of another family member. These results indicated that a worker's work-family conflict has no impact on their partner's nor on their adolescent children's assessment of their own food-related life. Although these findings go against expectations, Vieira et al. (2016) has reported that an individual's WtoFC is more likely to affect their own outcomes rather than those of their partner. Moreover, Westman (2001) has stated that partner effects tend to be weaker than actor effects for the same variables, and that partner effects may be indirect effects mediated by other variables such as communication between partners.

Parents' WtoFC also showed no direct partner effect on their adolescent children's SWFoL. Previous research has shown that WtoFC has negative consequences in children; however, these studies have been conducted in samples of younger children (Vieira et al., 2016; Dinh et al., 2017; Yucel and Latshaw, 2020b). One plausible explanation for this null result may be the adolescents' high degree of tolerance for their parents' inter-role conflict, as shown in a previous study with Chilean adolescents (Kinkead et al., 2017). This acceptance may keep the adolescent's experiences in the food domain unaffected by their parents' inter-role conflict between work and home.

The lack of parent-child partner effects in this regard may also be due to differences in food preferences and what SWFoL means for parents and adolescents. Researchers have reported that adolescents associate SWFoL with healthy and unhealthy foods (e.g., “junk food,” french fries), while mothers and fathers associate SWFoL mainly with healthy food (Schnettler et al., 2020a). Moreover, while this study's sample showed a high frequency of consumption of home-made foods per week, eating habits from these families also included consumptions of unhealthy (tasty) meals from other sources (i.e., ready-to-eat foods, delivery, restaurants, fast foods). These latter, less healthful eating habits have been associated with higher job demands in workers and working parents (Djupegot et al., 2017; Liu et al., 2017), but may be advantageous for adolescents' increasing food autonomy. Although parents still typically provide foods and are responsible for mealtimes during adolescence (Piccoli et al., 2017; Patel et al., 2018; Fleary and Ettienne, 2019), this life stage is characterized by the elaboration of identity and it is a time associated with increasing autonomy, when individuals want to make their own decisions, including what and when to eat (Piccoli et al., 2017).

Other partner effects showed that one parent's enjoyment of family meals does not contribute to their partner's assessment of their food-related life. This enjoyment also has no impact on the adolescent's assessment either, but only in the case of fathers, whereas it does have an effect when it comes from the mother. That is, although the relationship between the mothers' WtoFC and their adolescent children's perception of the atmosphere of family meals was of low strength, mothers seem to have a larger influence on their children's eating habits, compared to fathers. This influence might be explained by factors related to gendered parental roles and associated conditions. In this sample, a higher proportion of mothers had paid part-time jobs (40.8%), while most fathers worked full-time (72.3%). Work schedule has been associated with more frequent family meals (Sharif et al., 2017; Jones, 2018), and working part-time may make it more likely for mothers than fathers to share meals with their children. Mothers may also get more emotionally involved in their children's lives (Kinkead et al., 2017). Moreover, in Latin American countries, mothers are attributed larger responsibilities than fathers on food-related tasks (Persson Osowski and Mattsson Sydner, 2019; Schnettler et al., 2021b), and on child discipline around mealtimes (Schnettler et al., 2021b). Hence, even if the father is present for family meals, mothers take a more prominent role that affords them greater interactions with their children, increasing their SWFoL.

The last partner effects tested showed that parent's WtoFC was not associated with the other parent's perception of the atmosphere of family meals. This null effect aligns with previous studies showing no significant partner effects between both members of a couple's WtoFC and some outcomes (Yucel and Latshaw, 2020a). This result is also consistent with the lack of actor effects from both parent's WtoFC to their own perception of the atmosphere of family meals, as it has been suggested that if actor effects are not significant, it is likely that partner effects do not exist (Garcia et al., 2015). On the other hand, negative low strength partner effects were found for WtoFC and AFM in the mother-child dyad, but not in the father-child dyad, meaning that only the mother's work strains crossover to their children's experience of family meals.

In summary, the relationships found show low- and medium- strength relationships between the work-family interface and the food domain, and these relationships vary among family members. Results show that parents' WtoFC is not associated with their own perception of family meal atmosphere, but WtoFC has a low strength link to their own SWFoL. Low strength relationships were also found from the mothers' atmosphere of family meals to adolescents' SWFoL, and from mothers' WtoFC to adolescents' SWFoL *via* adolescents' perception of family meal atmosphere. Lastly, a medium-strength relationship was observed from the three family members' atmosphere of family meals to their own SWFoL, consistent with the expectation that this family event can contribute to the individual's assessment of their food domain.

Overall, in terms of the influence among family members, these effects indicate mothers' work-to-family conflict has a higher influence on their adolescent children's food domain than fathers'. The effect of the mother's WtoFC on the adolescent's perception of the atmosphere of family meals may be related to the greater responsibility for family meals assigned to women (Persson Osowski and Mattsson Sydner, 2019; Schnettler et al., 2021b). Mother-child interactions around food may include bickering about food choices, and food-related parenting practices exerted by mothers to promote a healthy diet and prevent their adolescent children's overweight and obesity (Martin-Biggers et al., 2014; Schnettler et al., 2021a). These arguments around food may be more severe when mothers experience WtoFC (Matias and Recharte, 2021).

### The Mediating Role of the Perception of the Atmosphere of Family Meals

Perception of the atmosphere of family meals was tested as a mediator between the parent's WtoFC and the three family members' SWFoL. No actor effects were found for either parent, while partner effects —in keeping with results from previous hypotheses— were only found from mothers' WtoFC and their adolescent children's SWFoL.

On the other hand, partner effects were found. Mothers' WtoFC was associated with the adolescent's SWFoL *via* the adolescent's perception of the atmosphere of family meals. This result supports that one parent's negative work experiences can have an indirect influence on their children's well-being (Matias and Recharte, 2021). Our findings expand on this knowledge by showing an indirect negative impact of the mother's work-to-family conflict on their children's well-being in the food domain. These effects may be explained by a conflictive mother-child relationship during family meals, based on the greater responsibility assigned to mothers than to fathers —for whom there was no mediating role— in terms of conducting family meals (Persson Osowski and Mattsson Sydner, 2019; Schnettler et al., 2021b). This indirect crossover from mother to adolescent may also contribute to explain the lower scores obtained by the adolescents in the SWFoL in comparison to their parents.

### Limitations

The first limitation of this study is its cross-sectional design. Although the APIM refers to effects, the relationships shown by these models are associations. Further studies with quasi-experimental and longitudinal designs can help test the potential causal relationships that we have proposed here. For instance, quasi-experiments would allow to compare differential effects of parents' family-to-work conflict levels (e.g., high vs. low) on family meal atmosphere and satisfaction with food-related life; a longitudinal quasi-experiment might help detect time order, that is, that the proposed dependent variables are indeed antecedents for the dependent variables (i.e., WtoFC → AFM, AFM → SWFoL) from one family member to another. A second limitation is that the sample was non-probabilistic. This sample was representative of the socioeconomic status in Chilean families [Asociación de Investigadores de Mercado (AIM), 2016], but not in terms of age, number of family members nor children per family. The latter two variables had a higher average in this sample than in the general population (family members: 4.4 and 2.2, respectively, and children per family: 3.1 and 1.3, respectively, INE [Instituto Nacional de Estadísticas (INE), 2018]. Therefore, these findings may not apply to families with different compositions to the one included in this study. Furthermore, data was self-reported, and responses might be biased due to social desirability. The third limitation is that questions regarding the household (family composition, the frequency of family meals, the source of family meals during a week—e.g., homemade foods, fat-food outlets—and the number of hours per day that they, their male partner and another person spent cooking during the week and on weekends) may present an incomplete perspective in this regard because these questions were answered only by mothers. The fourth limitation is the gender of the respondent adolescent was not considered in this analysis. Lastly, the Latin American context presents specific characteristics in terms of gender equality, family structure, and working conditions. Future research should account for these conditions and therefore include quasi-experimental and longitudinal designs and probabilistic sampling, asking fathers and adolescents demographic characteristics as well as eating and coking habits, measuring the possible different influence that parent may have on female and male adolescents and, conducting cross-cultural analysis to better understand the work-family interface, gender dynamics, and food-related life in different sociocultural contexts.

### Implications and Future Research

The mothers' link to their adolescents' food outcomes show that mothers still carry more responsibilities than fathers for the well-being of their children; this is the basis of the “double shift” that mothers are expected to fulfill, first at work and then at home, often to the detriment of their own well-being. This knowledge can inform employers and workplace policies, as considering the gendered dynamics of the work-home interface can help foster a culture that supports an egalitarian involvement of fathers in the home and childcare. Moreover, workplace policies focused on promoting the well-being of workers should include flexible schedules, sufficient time for mealtimes at work, and additional support to decrease work stress and overtime, among others.

These results suggest further research directions. First, given that the parents' and adolescents' perception of atmosphere of family meals has a positive influence on their own satisfaction with food-related life, despite parents' WtoFC, research should assess the underlying protective mechanisms that keep parents' negative work experiences from affecting family meals. The results of this study also suggest that future research should consider factors that explain the double effect of mothers on their children's well-being, that is, how in parallel the mother's perception of the atmosphere of family meals positively influences their adolescent children SWFoL, while the mothers' WtoFC negatively influence the same outcome through a negative effect on the adolescents' perception of the atmosphere of family meals.

## Data Availability Statement

The raw data supporting the conclusions of this article will be made available by the authors, without undue reservation.

## Ethics Statement

The studies involving human participants were reviewed and approved by Comité Ético Científico de la Universidad de La Frontera (Scientific Ethics Committee of the University of La Frontera). Written informed consent to participate in this study was provided by the participants' legal guardian/next of kin.

## Author Contributions

BSch designed the research study and wrote the first draft of the manuscript. BSch and LO conducted the research and revised manuscript drafts. HP handled and revised the database. EM-Z analyzed the data. EM-Z, GL, CA-B, and ML provided a critical analysis of the study throughout all stages. KB provided additional support for manuscript revision. All authors approved the manuscript in its final form.

## Funding

This study was funded by Fondecyt Project no. 1190017.

## Conflict of Interest

The authors declare that the research was conducted in the absence of any commercial or financial relationships that could be construed as a potential conflict of interest.

## Publisher's Note

All claims expressed in this article are solely those of the authors and do not necessarily represent those of their affiliated organizations, or those of the publisher, the editors and the reviewers. Any product that may be evaluated in this article, or claim that may be made by its manufacturer, is not guaranteed or endorsed by the publisher.
