# Supplementary material for: Relationships Between Work-to-Family Conflict and the Food Domain for Dual-Earner Parents With Adolescent Children
Source: Front Psychol. 2021 Dec 16;12:752209. doi: 10.3389/fpsyg.2021.752209 (PMC8716810; doi:10.3389/fpsyg.2021.752209)
Supplement: Supplementary file 1 [file Table_1.DOCX]

**Supplemental material**

**Supplemental Table 1.** Dyadic measurement invariance for the Work-to-Family Conflict (WtoFC) measure among mothers and fathers.

| **Model** | **χ^2^** | **df** | **p** | **D_χ^2^** | **D_df** | **RMSEA** | **D_RMSEA** | **CFI** | **D_CFI** | **TLI** |
| --- | --- | --- | --- | --- | --- | --- | --- | --- | --- | --- |
| 0 Configural (correlacted factors) | 28.902 | 19 | 0.068 | - | - | 0.033 | - | 0.994 | - | 0.991 |
| 1 Configural (correlated error terms) | 25.821 | 15 | 0.040 | 3.081 | 4 | 0.039 | 0.006 | 0.994 | 0.000 | 0.988 |
| 2 Metric (loadings fixed) | 29.552 | 18 | 0.042 | 3.731 | 3 | 0.037 | -0.002 | 0.993 | -0.001 | 0.987 |
| 3 Scalar (loadings and thresholds fixed) | 36.854 | 34 | 0.338 | 7.302 | 16 | 0.013 | -0.024 | 0.998 | 0.005 | 0.999 |

χ2 = Chi square.

df = Degrees of freedom.

D_χ2 = Delta of Chi-square.

D_df = Delta of degrees of freedom.

RMSEA = Root Mean Square Error of Approximations.

D_RMSEA = Delta of Root Mean Square Error of Approximations

CFI = Comparative Fit Index.

D_CFI = Delta of Comparative Fit Index

TLI = Tuker-Lewis Index.

**Supplemental Table 2.** Dyadic measurement invariance for the Project-EAT Atmosphere of family meals (AFM) scale among mothers and fathers.

| **Model** | **χ^2^** | **df** | **p** | **D_χ^2^** | **D_df** | **RMSEA** | **D_RMSEA** | **CFI** | **D_CFI** | **TLI** |
| --- | --- | --- | --- | --- | --- | --- | --- | --- | --- | --- |
| 0 Configural (correlacted factors) | 107.869 | 19 | 0.000 | - | - | 0.099 | - | 0.958 | - | 0.937 |
| 1 Configural (correlated error terms) | 81.387 | 15 | 0.000 | 26.482 | 4 | 0.097 | -0.002 | 0.968 | 0.010 | 0.941 |
| 2 Metric (loadings fixed) | 76.569 | 18 | 0.000 | 4.818 | 3 | 0.083 | -0.014 | 0.972 | 0.004 | 0.957 |
| 3 Scalar (loadings and thresholds fixed) | 57.811 | 38 | 0.021 | 18.758 | 20 | 0.033 | -0.050 | 0.991 | 0.019 | 0.993 |

χ2 = Chi square.

df = Degrees of freedom.

D_χ2 = Delta of Chi-square.

D_df = Delta of degrees of freedom.

RMSEA = Root Mean Square Error of Approximations.

D_RMSEA = Delta of Root Mean Square Error of Approximations

CFI = Comparative Fit Index.

D_CFI = Delta of Comparative Fit Index

TLI = Tuker-Lewis Index.

**Supplemental Table 3.** Dyadic measurement invariance for the Satisfaction with Food-related life (SWFoL) scale among mothers and fathers.

| **Model** | **χ^2^** | **df** | **p** | **D_χ^2^** | **D_df** | **RMSEA** | **D_RMSEA** | **CFI** | **D_CFI** | **TLI** |
| --- | --- | --- | --- | --- | --- | --- | --- | --- | --- | --- |
| 0 Configural (correlacted factors) | 103.187 | 34 | 0.000 | - | - | 0.066 | - | 0.974 | - | 0.965 |
| 1 Configural (correlated error terms) | 64.205 | 29 | 0.000 | 38.982 | 5 | 0.051 | -0.015 | 0.987 | 0.013 | 0.979 |
| 2 Metric (loadings fixed) | 65.666 | 33 | 0.000 | 1.461 | 4 | 0.046 | -0.005 | 0.988 | 0.001 | 0.983 |
| 3 Scalar (loadings and thresholds fixed) | 166.293 | 58 | 0.021 | 100.627 | 25 | 0.063 | 0.017 | 0.959 | -0.029 | 0.968 |

χ2 = Chi square.

df = Degrees of freedom.

D_χ2 = Delta of Chi-square.

D_df = Delta of degrees of freedom.

RMSEA = Root Mean Square Error of Approximations.

D_RMSEA = Delta of Root Mean Square Error of Approximations

CFI = Comparative Fit Index.

D_CFI = Delta of Comparative Fit Index

TLI = Tuker-Lewis Index.

**Supplemental Table 4.** Dyadic measurement invariance for the Project-EAT Atmosphere of family meals (AFM) scale among mothers and adolescent children.

| **Model** | **χ^2^** | **df** | **p** | **D_χ^2^** | **D_df** | **RMSEA** | **D_RMSEA** | **CFI** | **D_CFI** | **TLI** |
| --- | --- | --- | --- | --- | --- | --- | --- | --- | --- | --- |
| 0 Configural (correlacted factors) | 61.714 | 19 | 0.000 | - | - | 0.069 | - | 0.977 | - | 0.967 |
| 1 Configural (correlated error terms) | 47.974 | 15 | 0.000 | 13.740 | 4 | 0.068 | -0.001 | 0.983 | 0.006 | 0.967 |
| 2 Metric (loadings fixed) | 48.090 | 18 | 0.000 | 0.116 | 3 | 0.059 | -0.009 | 0.984 | 0.001 | 0.975 |
| 3 Scalar (loadings and thresholds fixed) | 105.032 | 38 | 0.021 | 56.942 | 20 | 0.061 | 0.002 | 0.964 | -0.020 | 0.974 |

χ2 = Chi square.

df = Degrees of freedom.

D_χ2 = Delta of Chi-square.

D_df = Delta of degrees of freedom.

RMSEA = Root Mean Square Error of Approximations.

D_RMSEA = Delta of Root Mean Square Error of Approximations

DFI = Comparative Fit Index.

D_CFI = Delta of Comparative Fit Index

TLI = Tuker-Lewis Index.

**Supplemental Table 5.** Dyadic measurement invariance for the Satisfaction with Food-related life (SWFoL) scale among mothers and adolescent children.

| **Model** | **χ^2^** | **df** | **p** | **D_χ^2^** | **D_df** | **RMSEA** | **D_RMSEA** | **CFI** | **D_CFI** | **TLI** |
| --- | --- | --- | --- | --- | --- | --- | --- | --- | --- | --- |
| 0 Configural (correlacted factors) | 102.526 | 34 | 0.000 | - | - | 0.065 | - | 0.973 | - | 0.964 |
| 1 Configural (correlated error terms) | 96.227 | 29 | 0.000 | 6.299 | 5 | 0.070 | 0.005 | 0.974 | 0.001 | 0.959 |
| 2 Metric (loadings fixed) | 106.873 | 33 | 0.000 | 10.646 | 4 | 0.069 | -0.001 | 0.971 | -0.003 | 0.960 |
| 3 Scalar (loadings and thresholds fixed) | 505.756 | 58 | 0.021 | 398.883 | 25 | 0.128 | 0.059 | 0.822 | -0.149 | 0.863 |

χ2 = Chi square.

df = Degrees of freedom.

D_χ2 = Delta of Chi-square.

D_df = Delta of degrees of freedom.

RMSEA = Root Mean Square Error of Approximations.

D_RMSEA = Delta of Root Mean Square Error of Approximations

CFI = Comparative Fit Index.

D_CFI = Delta of Comparative Fit Index

TLI = Tuker-Lewis Index.

**Supplemental Table 6.** Dyadic measurement invariance for the Project-EAT Atmosphere of family meals (AFM) scale among fathers and adolescent children.

| **Model** | **χ^2^** | **df** | **p** | **D_χ^2^** | **D_df** | **RMSEA** | **CFI** | **D_CFI** | **TLI** |
| --- | --- | --- | --- | --- | --- | --- | --- | --- | --- |
| 0 Configural (correlacted factors) | 67.402 | 19 | 0.000 | - | - | 0.073 | 0.972 | - | 0.958 |
| 1 Configural (correlated error terms) | 56.170 | 15 | 0.000 | 11.232 | 4 | 0.076 | 0.976 | 0.004 | 0.955 |
| 2 Metric (loadings fixed) | 58.305 | 18 | 0.000 | 2.135 | 3 | 0.069 | 0.976 | 0.000 | 0.963 |
| 3 Scalar (loadings and thresholds fixed) | 98.413 | 38 | 0.000 | 40.108 | 20 | 0.058 | 0.965 | -0.011 | 0.974 |

χ2 = Chi square.

df = Degrees of freedom.

D_χ2 = Delta of Chi-square.

D_df = Delta of degrees of freedom.

RMSEA = Root Mean Square Error of Approximations.

D_RMSEA = Delta of Root Mean Square Error of Approximations

CFI = Comparative Fit Index.

D_CFI = Delta of Comparative Fit Index

TLI = Tuker-Lewis Index.

**Supplemental Table 7.** Dyadic measurement invariance for the Satisfaction with Food-related life (SWFoL) scale among fathers and adolescent children.

| **Model** | **χ^2^** | **df** | **p** | **D_χ^2^** | **D_df** | **RMSEA** | **D_RMSEA** | **CFI** | **D_CFI** | **TLI** |
| --- | --- | --- | --- | --- | --- | --- | --- | --- | --- | --- |
| 0 Configural (correlacted factors) | 86.531 | 34 | 0.000 | - | - | 0.057 | - | 0.975 | - | 0.967 |
| 1 Configural (correlated error terms) | 75.241 | 29 | 0.000 | 11.290 | 5 | 0.058 | 0.001 | 0.978 | 0.003 | 0.966 |
| 2 Metric (loadings fixed) | 91.996 | 33 | 0.000 | 16.755 | 4 | 0.061 | 0.003 | 0.972 | -0.006 | 0.962 |
| 3 Scalar (loadings and thresholds fixed) | 202.598 | 58 | 0.000 | 110.602 | 25 | 0.073 | 0.012 | 0.932 | -0.040 | 0.947 |

χ2 = Chi square.

df = Degrees of freedom.

D_χ2 = Delta of Chi-square.

D_df = Delta of degrees of freedom.

RMSEA = Root Mean Square Error of Approximations.

D_RMSEA = Delta of Root Mean Square Error of Approximations.

CFI = Comparative Fit Index.

D_CFI = Delta of Comparative Fit Index

TLI = Tuker-Lewis Index.
